# A Rare Case of Double True Umbilical Cord Knots With a Nuchal Cord: A Case Report

**DOI:** 10.7759/cureus.89069

**Published:** 2025-07-30

**Authors:** Sylvia Dygulski, Riah S Lee, Melissa Lin, Nidhi Chawla, Mason Hui, Sadia Sahabi

**Affiliations:** 1 Surgery, Touro College of Osteopathic Medicine, Middletown, USA; 2 Obstetrics and Gynecology, Touro College of Osteopathic Medicine, Middletown, USA; 3 Medicine and Surgery, Touro College of Osteopathic Medicine, Middletown, USA; 4 Minimally Invasive Gynecologic Surgery, and Obstetrics and Gynecology, Overlook Medical Center, Summit, USA; 5 Obstetrics and Gynecology, Vassar Brothers Medical Center, Poughkeepsie, USA

**Keywords:** double true umbilical cord knot, false umbilical cord knot, nuchal cord, true umbilical cord knot, umbilical cord knot, umbilical cord true tight knot

## Abstract

A double true umbilical cord knot (TUCK) is a rare complication of pregnancy that is often missed on ultrasonography. The stricture caused by TUCK can lead to occlusion of fetal circulation, fetal asphyxia, and subsequent death. Despite these risks, there is a lack of evidence and no specific consensus on both antepartum and intrapartum management of TUCK. We report a rare case of a healthy male neonate with a double TUCK and nuchal cord delivered vaginally with a vacuum assist.

A 26-year-old female, G2P1001, at 39 weeks of gestation, presented to the labor and delivery unit for induction of labor for suspected fetal macrosomia. The labor course was complicated by recurrent variable decelerations during the second stage of labor, which prompted an assisted vaginal delivery. Upon delivery, a single tight nuchal cord was noted, which was successfully reduced. Further examination revealed two TUCKs. Subsequently, the umbilical cord was doubly clamped and cut, and the neonate was transferred to the NICU team for further evaluation. Neonatal and maternal outcomes were reassuring, with no immediate complications observed.

The umbilical cord plays an essential role in transporting nutrients and oxygen to the fetus and discarding waste products. Polyhydramnios, small fetus size, gestational DM, and male fetuses have been reported to be associated with an increased risk of TUCK. Despite advancements in prenatal imaging, true knots remain difficult to detect until delivery. If TUCK remains undiagnosed until delivery, it increases the likelihood of fetal compromise and death. Therefore, close surveillance is warranted in these unusual cases. This case highlights the critical need for monitoring throughout pregnancy, allowing for timely intervention and optimization of maternal and neonatal outcomes.

The coexistence of double TUCK and nuchal cord is a rare complication that is difficult to detect prenatally due to nonspecific imaging findings. In light of the lack of evidence-based treatment options available, we highlight the importance of further research for an accurate prenatal diagnosis of TUCK and to reduce complications and fetal demise.

## Introduction

A double true umbilical cord knot (TUCK) is a rare complication of pregnancy that is often missed on ultrasonography. A single TUCK is seen in 0.3% to 1.2% of pregnancies [1]. The incidence of a double TUCK alone is less than 0.1%, making the incidence of a double TUCK in addition to a nuchal cord even rarer [1]. With an adequate amount of amniotic fluid, TUCKs can be detected via ultrasonography [2]. However, recent studies suggest that only 12% of umbilical cord knot cases are accurately diagnosed antepartum using 2D ultrasonography [3]. Despite its low sensitivity, the stricture caused by TUCK can lead to occlusion of fetal circulation, fetal asphyxia, and subsequent death. True umbilical cord knots have been reported to be associated with a four- to 10-fold increase in the risk of fetal demise [4]. Currently, there is a lack of evidence and no specific consensus on both antepartum and intrapartum management of TUCK. We present a rare case of a healthy male neonate with a favorable outcome following a vaginal delivery, despite an incidental finding of TUCK and a nuchal cord.

## Case presentation

A 26-year-old female, G2P1+1, at 39 weeks of gestation, presented to the labor and delivery unit for induction of labor for suspected fetal macrosomia. Fetal weight was estimated to be in the 96th percentile at 36 weeks of gestation. The maternal prenatal course was notable for obesity, nicotine usage, and a history of persistent nausea and vomiting. Upon initial examination, the patient’s vital signs were as follows: temperature 98.3°F, heart rate 80 bpm (regular), respiratory rate 18 breaths per minute, blood pressure 120/76 mmHg, and a weight of 107.2 kg. The patient's relevant lab work is included in Table 1.

**Table 1:** Blood test results Hgb: Hemoglobin, Hct: Hematocrit, Plt: Platelet

Parameters	Results	Reference range
WBC	9.0	3.5 - 10.0 x 10^9^/L
Hgb	10.4	12.0-16.0 g/dL
Hct	30.2	35.0-46.0 %
Plt	152	150-400 x 10^9^/L

**Figure 1:**
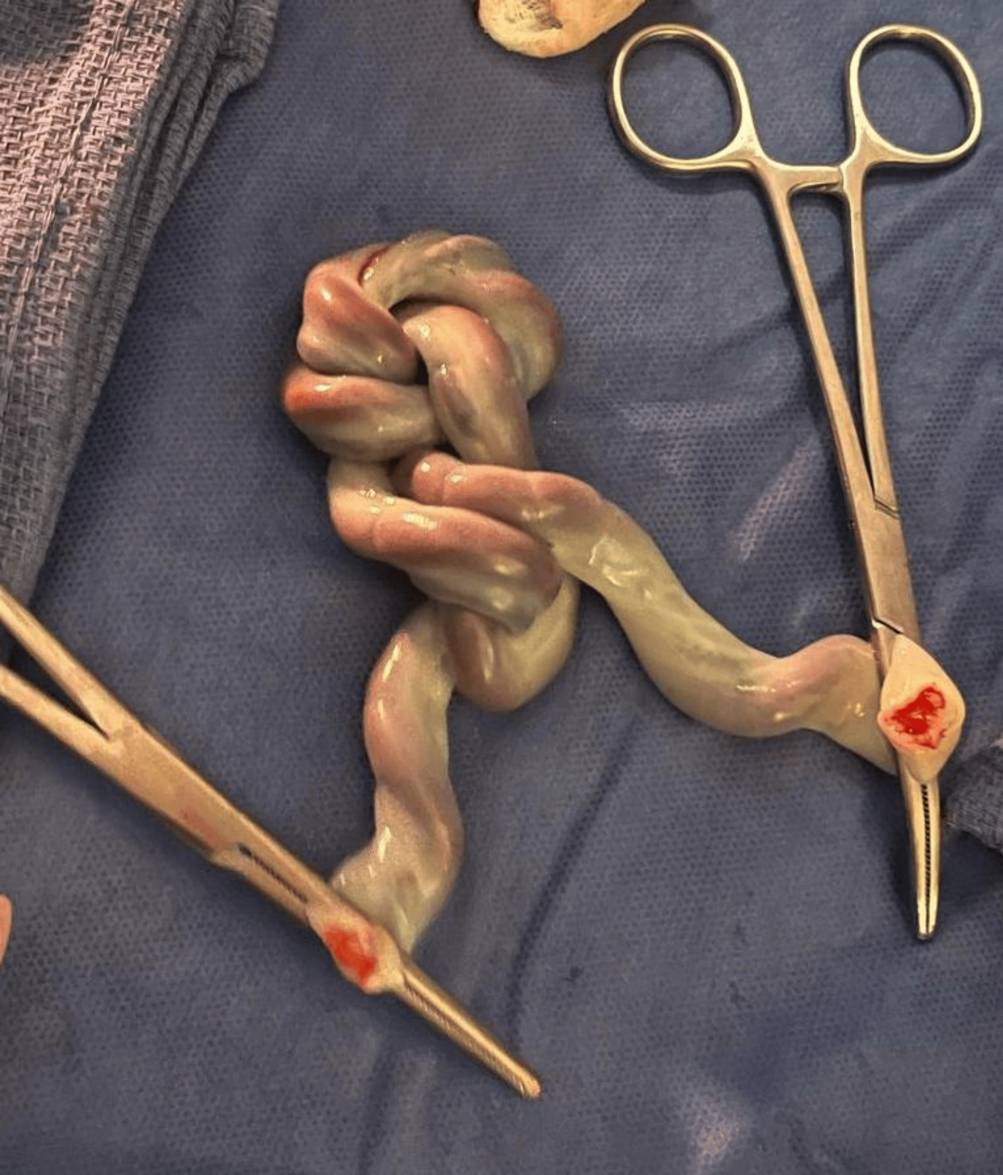
Double TUCK without signs of significant constriction TUCK: True umbilical cord knot

The patient's first pregnancy resulted in an uncomplicated vaginal delivery six years prior at 42 weeks of gestation. In this second pregnancy, the patient's labor was induced with a combination of mechanical dilation using the Cook® catheter (Cook Medical, Bloomington, IN, USA), misoprostol, and oxytocin augmentation. Artificial rupture of membranes revealed clear amniotic fluid. During the second stage of labor, recurrent variable decelerations were noted, which prompted closer monitoring. On vaginal examination, the fetal head was at the +2 station and advanced to +3 with maternal pushing. Given the concerning fetal heart tracings and prolonged expulsive efforts, the patient was counseled on an operative vaginal delivery. A vacuum-assisted vaginal delivery was performed with a single application and one successful vacuum application by the physician, which resulted in atraumatic delivery of the fetal head. Upon delivery, a single tight nuchal cord was identified and was successfully reduced. Further examination revealed two true knots in the umbilical cord, which were intact without evidence of significant constriction (Figure 1).

Subsequently, the umbilical cord was doubly clamped and cut. On neonatal exam, the appearance, pulse, grimace, activity, and respiration (APGAR) scores of 8 and 9 were recorded at one and five minutes, respectively, and initial cord gases were found to be within normal limits. No other fetal anomaly was noted. Neonatal outcomes and maternal outcomes were reassuring, with no immediate complications observed. However, given the incidental finding of two TUCKs and a nuchal cord at delivery, the neonate was evaluated by the neonatal intensive care unit (NICU) team as a standard precaution.

Intravenous Pitocin was initiated for postpartum hemorrhage prophylaxis. The placenta was delivered spontaneously approximately seven minutes later. Postpartum evaluation showed minimal maternal bleeding and bilateral labial tears, which were repaired with absorbable 3-chromic sutures. Despite the findings of a double TUCK and a nuchal cord, the neonate demonstrated no signs of distress or hypoxia. To date, no further complications have been reported.

## Discussion

The umbilical cord is composed of blood vessels that play a crucial role in the transfer of nutrients and oxygen from the mother to the fetus. Given its profound role in fetal development, any abnormalities to the cord can lead to detrimental outcomes, such as intrauterine growth restriction and stillbirth [5]. One such abnormality is a TUCK, a rare complication of an umbilical cord twisting on itself and forming a knot [1]. The occurrence of a double TUCK is reported to be less than 0.1% [6]. In comparison, a false umbilical knot, known as a 'pseudoknot,' is formed by the localized protuberance of Wharton’s jelly along the umbilical cord [7]. A false umbilical cord knot is much more common and does not lead to adverse clinical outcomes as TUCKs do. 

While the diagnosis of two TUCKs is rare, the coexistence of a double TUCK in addition to a nuchal cord is extremely uncommon. A nuchal cord occurs when the umbilical cord wraps around the neck of the fetus [8]. While most single nuchal loops are benign, a tight nuchal cord can increase the risk of asphyxia or even fetal demise [5]. The reported incidence of a single loop ranges from 24% to 28%, whereas multiple loops of nuchal cord are much rarer, occurring in 0.5% to 3.3% of pregnancies [9].

The formation of a TUCK is multifactorial, with reported risk factors including polyhydramnios, male fetus, a long umbilical cord, excessive fetal movement, prior amniocentesis, advanced maternal age, multiparity, and diabetes mellitus [10,11]. While TUCKs frequently occur without clinical significance, they can pose significant risks to fetal well-being, including hypoxia, fetal distress, and, in rare cases, intrauterine fetal demise [12]. Post-delivery complications associated with TUCKs include hypoglycemia and neonatal jaundice [13]. In our case, despite the presence of both double TUCK and a tight nuchal cord, the neonate demonstrated no signs of distress or hypoxia, as evidenced by APGAR scores of 8 and 9 at one and five minutes, respectively, with normal cord gases. We surmise that the lack of significant umbilical cord constriction most likely contributed to the favorable fetal and maternal outcomes.

Given the detrimental outcomes associated with TUCKs, early prenatal detection is essential. Unfortunately, they are often undetected prenatally due to the asymptomatic nature of the condition and the lack of a definitive in-utero ultrasound sign. The inability to accurately evaluate the full length of the umbilical cord prenatally further adds to the diagnostic challenge. However, with the advancement of 3D high-definition (HD) flow and color Doppler, prenatal diagnosis of TUCK has become more feasible [7]. Some of the patterns found on the ultrasound include the 'hanging noose sign' as well as a 'four-leaf clover sign' [14]. Notably, 3D HD flow, or the color Doppler, is typically indicated after clinical suspicion arises with inconclusive 2D imaging. With a detection rate of 63.2%, 3D HD flow demonstrates a significantly higher sensitivity compared to existing modalities such as 2D and color Doppler ultrasounds [15]. Nevertheless, TUCKs often remain undetected prenatally, even after the use of advanced imaging, and are frequently identified incidentally at delivery. 

While these imaging modalities are far from perfect, they highlight the possibility for early detection of TUCK and the importance of vigilant antepartum and intrapartum monitoring to reduce the risk of unforeseen maternal or fetal distress. Early detection of both TUCK and nuchal cords using Doppler technology will enable clinicians to implement tailored management strategies, thereby minimizing unfavorable neonatal complications. 

## Conclusions

The coexistence of double TUCK and nuchal cord is a rare presentation that poses diagnostic challenges due to its nonspecific imaging findings and variable symptomatic presentations. This report presents a rare, incidental finding of both double TUCK and a nuchal cord with a favorable outcome. However, it underscores the importance of advancing prenatal diagnostic tools to reduce associated complications.
